# Using Mixed Methods Integration to Evaluate the Structure of Help-Seeking Barriers Scale: A Survivor-Centered Approach

**DOI:** 10.3390/ijerph19074297

**Published:** 2022-04-03

**Authors:** Karen Birna Thorvaldsdottir, Sigridur Halldorsdottir, Denise M. Saint Arnault

**Affiliations:** 1 School of Health Sciences, University of Akureyri, 600 Akureyri, Iceland; sigridur@unak.is; 2 Department of Health Behavior and Biological Sciences, University of Michigan, Ann Arbor, MI 48109, USA; starnaul@umich.edu

**Keywords:** interpersonal trauma, gender-based violence, help-seeking barriers, mental health, trauma recovery, survivor-centered, cross-cultural adaptation, construct validation, mixed methods, integration

## Abstract

Despite the high prevalence of adverse health and trauma-related outcomes associated with intimate partner violence (IPV), help-seeking and service utilization among survivors is low. This study is part of a larger mixed-methods and survivor-centered validation study on the Icelandic Barriers to Help-Seeking for Trauma (BHS-TR) scale, a new barriers measure focused on trauma recovery. A mixed-methods legitimation strategy of integration was employed to evaluate the BHS-TR structure in samples of IPV survivors. The merging of qualitative (*n* = 17) and quantitative (*n* = 137) data through a joint display analysis revealed mainly complementarity findings, strengthening the scale’s overall trustworthiness and validity evidence. Divergent findings involved items about mistrust, perceived rejection, stigmatization, fearing vulnerability, and safeguarding efforts that were significant help-seeking barriers in the survivors’ narratives, whereas factor analysis indicated their removal. These BHS-TR items were critically evaluated in an iterative spiraling process that supported the barriers’ influence, illuminated core issues, and guided potential refinements. This work contributes to the growing field of mixed methods instrument validation placing equal status on qualitative and quantitative methods and emphasizing integration to provide more complete insights. Moreover, the study’s findings highlight the added value of further exploring divergence between two sets of data and the importance of giving attention to the voices of the target population throughout the validation process.

## 1. Introduction

### 1.1. Gender-Based Violence

Gender-based violence (GBV) is a public health crisis of pandemic proportions and one of the most prevalent human rights violations worldwide [1,2]. GBV refers to harmful acts directed against an individual because of their biological sex or gender identity. It is deeply rooted in gender inequality and cultural norms that uphold male domination and female subordination [2,3]. While people of all genders may experience GBV, women-identifying individuals are disproportionately impacted by it, and the most common form of GBV is intimate partner violence (IPV) against women [2,3,4]. IPV has been defined as any current or former intimate partner behavior that causes physical, sexual, or psychological harm, including physical aggression, sexual coercion, emotional abuse, and controlling actions [4]. Global estimates from the World Health Organization indicate that almost one-third (27%) of women have been subjected to violence by their intimate partners [1].

### 1.2. Violence against Women in the Nordic Countries

The Nordic countries are often considered the most gender-equal countries in the world [5,6], and according to the Global Gender Gap Index, Iceland had in 2021 closed 89.2% of its overall gender gap, holding the top spot for 12 years in a row [7]. While critical domains are used to measure gender equality (e.g., health and survival, educational access and attainment, economic participation, political power, and time allocation), these indices do not take GBV into account [5,6,7]. Previous research has reported a high prevalence of violence against women in the Nordic countries [8,9,10]. One of the most extensive studies in this regard (*n* = 42,000 women) was conducted by the European Union (EU) Agency for Fundamental Rights, which found lifetime prevalence rates of IPV in the Nordic countries to be among the highest of the 28 EU Member States. With an EU average of 22%, ranging between 13% and 32%, the rates were 28% in Sweden, 30% in Finland, and 32% in Denmark [11]. In Iceland (a non-EU member), a national survey estimated the lifetime prevalence of IPV against women at 22.4% [12].

### 1.3. Help-Seeking for Trauma Recovery

Numerous studies have documented the severe impact of IPV, a form of interpersonal trauma, on survivors’ health and well-being, showing increased risk of depression, post-traumatic stress disorder (PTSD), anxiety, somatic symptoms, substance abuse, and suicidal ideation [13,14,15,16]. This suffering is associated with functional impairment, low sense of coherence (SOC), and substantially reduced quality of life [17,18,19], even years after leaving the abusive relationship [20,21].

Despite these adverse outcomes related to IPV, previous research has shown that help-seeking among survivors is low in most countries. Some never seek help, and those who do mainly choose informal sources of help, usually from their family or friends and are less likely to seek formal help, such as from shelters, healthcare services, or the police [22,23,24,25]. It should be noted that a significant body of literature suggests that women with a history of IPV use healthcare services more than non-abused women, especially primary care and emergency departments [26,27,28]. Still, these women rarely disclose the violence and often do not receive the support and appropriate care they need [29,30,31].

The IPV help-seeking literature is primarily focused on escaping the violence and attending to the immediate harm caused. While these often first steps are critical, there is a need for an increased focus on survivors’ pathways for trauma recovery [32,33,34]. Help-seeking after IPV is a complex journey involving a series of meaning-making judgments and socially engaged and culturally informed actions [35,36,37], and the road to recovery is often challenging [38,39].

Findings of low help-seeking rates are consistent with other studies reporting that IPV survivors are faced with a wide range of barriers on sociocultural, structural, interpersonal, and individual levels, e.g., normalization of violence, access challenges, fearing consequences of disclosure, and self-blame [30,40,41,42]. Moreover, studies have indicated that survivors with depression, PTSD, and low SOC face even more significant barriers to help-seeking, such as symptom burden, fearing mental illness stigmatization, and a weak sense of manageability and meaning, making it more challenging to take action [21,36,43,44].

### 1.4. Use of Mixed Methods for Instrument Validation

In a widely used definition based on a review of definitions, mixed methods research is defined as a “type of research in which a researcher or team of researchers combines elements of qualitative and quantitative research approaches (e.g., use of qualitative and quantitative viewpoints, data collection, analysis, inference techniques) for the broad purposes of breadth and depth of understanding and corroboration [45] (p. 123)”. In the mixed methods research field, meta-inferences refer to inferences stemming from both qualitative (Qual) and quantitative (Quan) findings integrated into a coherent whole [46,47].

One of the earliest examples of using multiple research methods for validation dates to the 1950s, with Campbell and Fiske’s [48] framework giving rise to methodological triangulation and arguing that the convergence of findings derived from more than one method would strengthen the evidence of validity. However, as innovative and valuable their framework has been, it is first and foremost Quan. To date, in the instrument development literature, construct validation is often conceived as mainly a Quan endeavor [45,49,50,51,52]. When Qual data are used, it is usually only granted a supplementary role to Quan data, and often the methods are utilized in isolation rather than fully integrated [50,52,53]. Still, there is a growing literature on mixed methods validation. A few frameworks have been developed that place equal value on Quan and Qual methods, focusing on validity and trustworthiness, and emphasizing the integration or “mixing” of findings from both databases to inform validation evidence for a measure [49,51,52,54].

The “fit” of data integration refers to the coherence of Quan and Qual findings [55]. Such assessment is likely to lead to four possible outcomes: Confirmation is when the findings are consistent with each other, supporting drawing the same conclusion from each. Complementarity is when the findings tell different but nonconflicting stories (reflecting different sides of the same coin). Expansion is when the findings diverge to a certain degree but, when combined, can expand insights. Discordance is when the findings are inconsistent, contradictory, or disagree with each other [55,56].

The term legitimation [46] has been recommended to refer to validity and quality in mixed methods studies, as it considers both Quan and Qual research paradigms [47,57]. A legitimation typology was developed to guide researchers to ensure design quality and interpretive rigor to yield high-quality meta-inferences. The typology includes several legitimation types and is grounded on the notion that legitimation is not only an outcome but a process occurring throughout all stages of the research [46,47,57,58]. Recently a new type of legitimation was proposed to be added to the typology, “Divergent Findings Legitimation” [59], stemming from the challenge researchers face when Quan and Qual findings diverge [55,59,60,61]. Failure to follow up on divergence can threaten legitimation, and this new type is about the extent to which researchers explore and learn from such findings [59].

### 1.5. Validation of the Barriers to Help-Seeking for Trauma Scale

The Barriers to Help-Seeking for Trauma (BHS-TR) scale was developed from an existing mental health barriers measure [62] focusing on service use for mental disorders. Based on an international literature review about barriers to seeking help after trauma and findings from focus groups and individual interviews with American and Irish GBV survivors, the original scale was adapted for GBV survivorship [21,63]. The scale added new items about normalization, shame, mistrust, perceived rejection, fearing the consequences, and feeling frozen, making the measure more trauma-specific and survivor-centered. The BHS-TR consists of seven barrier subscales that can be grouped into two structural and internal barriers indices. The scale was psychometrically sound in a sample of American GBV survivors [63].

The BHS-TR was translated and cross-culturally adapted into the Icelandic language and context [64] and initially validated in a mixed-methods study among IPV survivors in Iceland [65], creating the first Icelandic trauma-specific measure that assesses help-seeking barriers. An essential part of this work was qualitatively evaluating the scale through cognitive interviewing, resulting in the development of new barrier items based on the survivors’ lived experiences. Using these findings, we utilized building to adapt the BHS-TR scale and then carried out a psychometric evaluation of the whole instrument with the additional items. Both Qual and Quan phases provided evidence that the Icelandic BHS-TR is relevant, reliable, and valid [64,65]. Nevertheless, there was a noticeable mismatch between the Qual and Quan findings regarding several items on the scale. Primarily, items that were significant barriers to help-seeking in the survivors’ narratives were problematic in the exploratory factor analysis technique, mainly due to cross-loadings with different factors. According to the factor analysis procedure, these cross-loaded items would be dropped and hereafter referred to as the dropped items [65]. This mismatch between the participants’ narratives and the factor analysis results points to a legitimation issue, demonstrating the need for further systematic assessment of the coherence of barriers to help-seeking Qual and Quan findings.

### 1.6. The Current Study

This study is part of a larger mixed-methods and survivor-centered validation study on the Icelandic BHS-TR scale. The more extensive study followed well-established international guidelines and best practices for cross-culturally adapting and validating measures [66,67,68]. An exploratory sequential mixed methods design [69] was used, conducted in two phases (Qual → Quan) with equal emphasis given to both phases. The primary aim of this present study was to use a mixed-methods legitimation strategy of integration to evaluate the BHS-TR structure by merging the Qual and Quan findings and examining meta-inferences on divergence.

Specifically, this study:

1. Integrated the Qual and Quan data to discover convergent (confirmation or complementarity) and divergent (expansion or discordance) validation evidence for the BHS-TR.
2. Critically evaluated divergent items by examining the following:
  - influence of the barriers on the survivors’ help-seeking,
  - factor cross-loadings for the most strongly endorsed items,
  - mean score differences for survivors’ subsamples (depression, PTSD, and SOC).
3. Made recommendations about the benefits of this integration to BHS-TR refinements.

## 2. Materials and Methods

### 2.1. Study Design, Integration, and Legitimation

This study used a mixed methods spiraling design [49,56,70] that employed integration strategies at the design, methods, interpretation, and reporting levels [55] through merging and joint display. We implemented nine types of legitimation strategies to ensure design quality and interpretive rigor to generate high-quality meta-inferences: sample integration, inside–outside (emic–etic), weakness minimization, sequential, commensurability approximation, multiple validities, integration, pragmatic, and divergent findings legitimation [57,58,59].

### 2.2. Data Collection

A sequential parallel sampling design [71] was used for the Qual and Quan phases. The inclusion criteria for both phases were to self-identify as a woman, be 18 years old or older, live in Iceland, speak Icelandic, and have experienced IPV. Since what constitutes IPV can vary from person to person, we let participants self-identify as survivors based on the definition and examples of types of IPV provided in the introductory material, including physical, sexual, emotional, financial, and cyber violence. Additionally, for ethical reasons and the participants’ security, they had to have been out of the abusive relationship for at least a year.

#### 2.2.1. Qualitative Phase

The Qual data were comprised of in-person cognitive interviews [72,73] with 17 Icelandic IPV survivors; a total of 25 interviews was conducted between August 2019 and January 2020. Participants were recruited using purposive sampling from survivors’ centers and services in North and South Iceland. During recruitment, attempts were made to select a diversity of IPV survivors. A detailed description of the procedures has been previously reported [64].

#### 2.2.2. Quantitative Phase

The Quan data were gathered using an anonymous online survey advertised through social media posts and flyers distributed at various services for violence survivors in Iceland, located all over the country. Participants accessed the survey by following a link hosted on the Icelandic Directorate of Equality’s website, and a voluntary response sampling was applied. Qualtrics, a secure online software package, was used to build the survey, including the BHS-TR scale, demographics, help-seeking history, mental health, and trauma-recovery related outcome measures (detailed below). The data were collected between February and October 2020, and our sample included 137 IPV survivors in Iceland. A detailed description of the procedures has been previously reported [65].

### 2.3. Participants

The Qual sample included 17 women IPV survivors in Iceland, ages 18 to 64 years (M = 37.4, SD = 12.2). Most of the women were employed (70.6%), with about half having received a university degree (52.9%), and most had children (70.6%). Most of our sample self-reported a current mental or physical diagnosis (64.7%), and all had been faced with physical, emotional, and social health effects because of their traumatic experiences. The women in the Qual sample had all sought help because of the violence, especially when leaving the abusive relationship, hence the recruitment strategy. However, when asked explicitly about seeking help for trauma recovery, only a few (35.3%) said they had done so, and about half of them had received mental healthcare at some point in their life (52.9%).

The Quan sample included 137 women IPV survivors in Iceland, ages 19 to 76 years (M = 40.7, SD = 11.7). Most of the women were employed (64.2%), with over half having received a university degree (59.9%), and most had children (76.7%). Most of our Quan sample self-reported a current mental or physical diagnosis (67.9%), and most had received mental healthcare at some point in their life (81.8%). When asked about needing help for trauma recovery in the last 12 months, 75.9% said that they had needed help in general, of which 26.3% listed as needing it most of the time or very necessary. Furthermore, 70.1% said they had especially required mental health treatment, of which 29.2% listed as needing it most of the time or very necessary. Nonetheless, almost half of the women (45.3%) reported not seeking the professional help they needed in the last 12 months. Further information on the characteristics of both Qual and Quan samples can be found in Table 1.

### 2.4. Measures

#### 2.4.1. Barriers to Help-Seeking for Trauma Scale

Help-seeking barriers were measured with the BHS-TR scale, which asks about barriers to seeking help for trauma recovery in the last 12 months. Respondents answer on a 4-point Likert scale (1 = “Did not influence me”, 2 = “Influenced me a little”, 3 = “Influenced me somewhat”, 4 = “Strongly influenced me”), with a higher total score indicating more barriers [63]. The current Icelandic version of the scale has 26 items divided into two indices (Structural and Internal Barriers), each of which has four subscales (see Supplementary Table S1). Initially, the scale included 41 items, and a list of the 15 dropped items can be found in Supplementary Table S2. The 26-item BHS-TR was found to have sound psychometric properties in our previous work [65]. There were no missing data for the BHS-TR, and the Cronbach’s α in this study was 0.87.

#### 2.4.2. Patient Health Questionnaire-8

Depression was assessed with the Patient Health Questionnaire-8 (PHQ-8) [74]. It is a widely used and valid measure consisting of eight items based on the DSM-IV diagnostic criteria for depression. PHQ-8 is identical to the PHQ-9 without the suicidal thoughts item, making it more suitable for general survey use. Respondents are asked to assess the frequency of symptoms in the past two weeks on a 4-point response scale from 0 (“Not at all”) to 3 (“Nearly every day”), with a total score range from 0 to 24. A clinical cut-off score of ≥10 has been recommended to indicate probable current depression [74,75]. There were no missing data for the PHQ-8, and the Cronbach’s α in this study was 0.87.

#### 2.4.3. Post-Traumatic Stress Disorder Checklist for DSM-5

Post-traumatic stress disorder was assessed with the PTSD Checklist for DSM-5 (PCL-5) [76]. It is a widely used and validated measure consisting of 20 items corresponding to the DSM-5 symptom criteria for PTSD. Respondents are asked to rate how bothered they have been by the symptoms in the past month on a 5-point response scale from 0 (“Not at all”) to 4 (“Extremely”), with a total score range from 0 to 80. A clinical cut-off score of ≥31 has been recommended to indicate probable current PTSD [76,77]. There were no missing data for the PCL-5, and the Cronbach’s α in this study was 0.96.

#### 2.4.4. Sense of Coherence Scale-13

SOC was measured with the shortened version of the Sense of Coherence Scale (SOC-13), also known as the Orientation to Life Questionnaire [78]. It comprises 13 items about how people view their lives, measuring SOC’s three main dimensions: comprehensibility, manageability, and meaningfulness. Respondents rate their level of agreement or disagreement on a 7-point semantic differential scale that has two anchoring responses tailored to each item. The total score range is from 13 to 91, with a higher score indicating a stronger SOC. It has been proposed that a low SOC corresponds to scores of 13–57, medium SOC corresponds to scores of 58–74, and a high SOC corresponds to scores of 75–91 [79]. The measure has been shown to be reliable and valid in multiple studies conducted across many cultures [80]. Five participants had missing values on the SOC-13, and the Cronbach’s α in this study was 0.85.

### 2.5. Ethical Considerations

The National Bioethics Committee in Iceland approved all procedures and materials (Qual phase: VSNb2019060009/03.01; Quan phase: VSNb2019090016/03.01), and the study was reported to the Icelandic Data Protection Authority (Qual phase: 19–119; Quan phase: 19–166). After receiving detailed information about the study, all participants voluntarily gave informed consent to participate. Written consent was obtained from the interview participants, while the survey participants provided their consent by answering the survey. All participants received a list of local referral resources with the introduction and after participation. Moreover, they were all offered support from a psychologist if difficult emotional reactions had emerged.

### 2.6. Data Analysis

The data analysis for this study occurred in two stages, each with several steps employed in an iterative spiraling process [49,56,70]. First, we integrated the Qual and Quan findings to assess the coherence of the scale structure. Based on those findings, the next stage focused on divergent findings at the dropped item level.

#### 2.6.1. Mixed Methods Analysis

Coherence of the findings was determined by merging [55] Qual and Quan findings using joint display analysis [81,82]. As noted, there are four possible outcomes of comparing data and drawing conclusions about the fit: Confirmation, Complementarity, Expansion, and Discordance [55,56]. Only the expansion and discordant items were explored in the second analytic stage.

#### 2.6.2. Examination of Expansion and Discordant Findings

The examination of the expansion or discordant dropped items was guided by reconciliation and additional validation strategies [59,60], where we re-analyzed existing data with a new perspective and evaluated these items. This analysis was driven by a critical examination of Quan data based on Qual findings. All statistical analyses were performed using the SPSS Statistics software package (Version 27.0; IBM Corp., Armonk, NY, USA, 2020), and significance was assessed at the *p* ≤ 0.05 level.

**Level of Influence.** We determined that higher mean scores were a metric indicating a stronger level of influence of items on survivors’ help-seeking. We, therefore, rank-ordered all the BHS-TR items by mean scores and examined the frequency of survivors who endorsed those items at 3 (“Influenced me somewhat”) or 4 (“Strongly influenced me”). Only items that met the threshold of a mean score above 2.00 were included. The strongly endorsed and dropped items were retained for further evaluation in the subsequent step.

**Cross-Loadings.** Dropped items that cross-loaded at or above 0.35 were compared with retained items to evaluate whether the dropped items were conceptually distinct. Cross-loaded items that were conceptually covered by a retained item were not included in further analysis. Conceptually distinct items were evaluated in the subsequent step.

**Known-Groups Validity.** We evaluated whether influential and conceptually distinct dropped items could differentiate between groups of survivors using independent sample *t*-tests. We created “probable depression”, “probable PTSD”, and “low SOC” subsamples by using the PHQ-8 clinical cut-off score of ≥10, the PCL-5 clinical cut-off score of ≥31, and the SOC-13 scores of 13–57. Based on the relevant literature (e.g., [21,36,43,44]) and results from prior studies on the BHS-TR [63,65], it was expected that survivors with depression, PTSD, and low SOC would, on average, score higher on the barrier items than survivors without depression or PTSD and with high SOC.

## 3. Results

### 3.1. Merging of Qualitative and Quantitative Findings

The joint displays linking the Qual and Quan findings are shown in Table 2 (Structural Barriers) and Table 3 (Internal Barriers), revealing evidence of complementarity, expansion, and discordance. To illuminate the lived experiences of the barriers, we chose exemplar quotations from the survivors, reported in the Qual column. The items referred to (using their original numbers) in the Quan column can be found in Supplementary Tables S1 and S2.

#### 3.1.1. Complementarity Evidence

Most of the Qual and Quan findings were congruent, reinforced one another, were deemed complementary, and strengthened the overall validation evidence of the BHS-TR. This complementarity applied to the BHS-TR Structural and Internal Barriers indices, as well as for the following specific factors of barriers: Financial Concerns, Unavailable/Not Helpful, External Constraints, Problem Management Beliefs, Frozen/Confused, and Shame. Therefore, these items were not evaluated in any subsequent analyses.

#### 3.1.2. Expansion Evidence

Two factors (Inconvenience and Discrimination) and one category (Reveals Weakness) were found to need expansion. First, the Inconvenience factor revealed in the Quan phase was missing a key inconvenience item identified in the Qual phase. That factor comprised of only two items, likely affecting the poor internal consistency, and needs to be expanded. Secondly, when combined, the Qual and Quan findings for the Discrimination factor indicates the need for further development and evaluation of these items in a more diverse sample. Moreover, the expansion should involve additional items specifically on the prejudice and stigmatization of GBV survivors. Thirdly, the Reveals Weakness category expanded into the Weakness/Vulnerability factor but was missing a vulnerability barrier that had been central in the survivors’ narratives. This new BHS-TR factor deserves continuing attention and better understanding of why the one dropped weakness item did not work on the factor.

#### 3.1.3. Discordance Evidence

One factor (Mistrust/Rejection) and one category (Safeguard Yourself) were revealed to be discordant. First, despite the Qual evidence of relevance, face validity, and content validity, the Quan phase showed that all the items on the Mistrust/Rejection factor should be dropped. The concept of mistrust for BHS-TR requires additional examination. Secondly, despite the support for three new items from the Qual phase in the Safeguard Yourself category, two of them were dropped in the Quan phase. The concept of safeguarding yourself requires additional examination.

#### 3.1.4. Integration Implications from Joint Display

Taken together, ten items that were dropped in the factor analysis belong to an expansion or discordant factor or category and need further analysis. Expansion is required for one inconvenience item (#9), all the discrimination items (#20, 21, and 23), and one weakness item (#38). Discordance was identified for all the mistrust items (#31, 32, and 33) and two safeguard items (#36 and 37). These items were focused on in the second analysis stage.

### 3.2. Examination of Expansion and Discordant Findings

#### 3.2.1. Level of Influence

The BHS-TR items above 2.00 were ranked by mean score (see Table 4). Of the ten expansion and discordance dropped items from the coherence joint displays, seven were above the 2.00 threshold and are indicated in bold. Of these items, one was in the top 10, and five were in the top 20. For most of these items, more than 50% of survivors reported the barrier as somewhat or strongly influencing them in not seeking help (range 35.8% to 62.8%).

**Integration Implications from Influence Analysis.** The inconvenience item and two of the discrimination items did not reach the mean influence 2.00 threshold and were therefore omitted from further analysis in this study. Two expansion items (Weakness #38 and Discrimination #23) and five discordance items (Mistrust #31, 32, and 33 and Safeguard #36 and 37) had mean influences over 2.00 and were included in subsequent analyses.

#### 3.2.2. Cross-Loadings

Table 5 shows cross-loadings onto BHS-TR factors for the seven influential dropped items. The specific inferences drawn from this analysis are also listed in the table. None of the items were conceptually similar to retained BHS-TR items.

**Integration Implications from Cross-Loading Analysis.** This analysis revealed issues with the wording of the safeguarding yourself items. We discovered that the concepts of feeling mistrustful and frozen/confused overlap. Similarly, there was a significant overlap between the feeling of revealing weakness and shame. The analysis further supported that the expansion of the discrimination factor needs a GBV specific items. These conceptual overlaps and cultural nuances need additional data gathering and analysis to understand. Because these items were all identified as conceptually distinct, all seven were kept for the final analysis step in this study.

#### 3.2.3. Known-Groups Validity

We used *t*-tests to compare the mean scores for the influential and conceptually distinct items for subgroups of probable depression, probable PTDS, and low SOC survivors (see Table 6). The mean scores for the mistrust and safeguard items were all significantly higher for the probable depression, probable PTSD, and low SOC groups. However, there were no significant differences in the mean scores for the weakness item, as all the survivors’ groups scored high on average. The mean score for the discrimination item was significantly higher for the depression group only.

**Integration Implications from Known-Groups Analysis**. Five out of the seven remaining items were statistically significant for the distress sub-groups and the low SOC subgroup. While the mistrust and safeguard yourself items need refinements, this analysis provided further validity evidence for these two concepts of barriers. The indications of ceiling effects for the weakness item might be related to its shame concept overlap, demonstrating the need for further evaluation. The performance of the discrimination item supports making it more specific about prejudice or discrimination related to GBV, which might enhance its ability to distinguish between different groups.

## 4. Discussion

This mixed-methods study presents the integration process of evaluating the structure of BHS-TR, the first trauma-specific measure on help-seeking barriers for GBV survivors in Iceland. Our study is part of a larger mixed-methods validation study [64,65] that places equal value on the Qual and Quan phases, focusing on credibility/trustworthiness and reliability/validity. Traditionally, if mixed methods are used, instrument development and validation designs place more emphasis on the Quan phase [49,69]. The merging of the Qual and Quan findings provided more complete insights into the construct validation evidence than could be gained by either alone. A majority of our Qual and Quan findings were complementarity, telling different but congruent stories, strengthening the overall legitimation evidence of the BHS-TR.

The added value of the integration also involved identifying our expansion and discordant findings. Few mixed methods studies examine details of divergence [59,60], and in the past, conflicting evidence between Qual and Quan data have often led researchers to overlook or dismiss Qual findings [83]. It is essential to acknowledge and respect the value of divergence as a possible generative of unanticipated insights [84]. Our survivor-centered approach applying an iterative spiraling process helped illuminate the core issues and inform potential refinements. At the end of this spiraling analysis, we identified seven dropped BHS-TR items that deserved further attention, representing barriers that had strongly influenced the survivors from seeking help yet are not covered by the scale. These items belonged to four critical factors/categories of barriers (Mistrust/Rejection, Safeguard Yourself, Reveals Weakness, and Discrimination) that still require work to accurately capture what they are supposed to be measuring and become BHS-TR subscales.

The findings showed that the mistrust items were conceptually overlapping with items related to feeling frozen and confused, indicating the need to rewrite these critical items about the notion that people would not understand them or could not be trusted to help them. These barriers are often impacted by past encounters and are therefore central to the survivors’ help-seeking experience [21,30,41]. Harmful responses (e.g., disbelieving, pathologizing, or blaming survivors) from the helpers and helping services are likely to prevent additional help-seeking [35,41,85,86]. Further, the need to expand the discrimination factor to capture the stigma and prejudice associated with GBV were discovered in this study, consistent with previous studies demonstrating that fear of stigmatization is a major barrier among survivors [30,32]. For example, the IPV Stigmatization Model [40] describes how three components of stigma, cultural, internalized, and anticipated, hinder help-seeking behaviors.

The perception of harmful responses from helpers was also related to our findings on the safeguarding items, highlighting the importance of trauma-informed responses and services. Wanting to protect oneself from further pain, facing one’s experience, and being re-traumatized were significant deterrents to help-seeking for the participants in this study. A recent systematic review among trauma survivors shows similar results [85]. However, we detected potential issues involving the items being either written too broadly or too specific. Additional analysis will reveal if the reworded items load, along with the third safeguard item currently belonging to the Weakness/Vulnerability subscale, onto a new Safeguarding Efforts subscale.

The weakness item was the most hindering barrier of the dropped items and was in the top 10 influence rank order for all BHS-TR items. We did not detect a significant problem with the wording. Yet, this item loaded onto the Weakness/Vulnerability and Shame factors, which are related but still conceptually distinct, at about the same strength. What might be causing this is how the item is more related to feeling weak by opening up while the other items focus on being seen as weak by others. Interestingly, this item performed worst in the final validity testing step, as it could not differentiate between any of the groups. On average, all the survivors scored high regardless of distress symptoms or their SOC. That might be related to how intertwined beliefs about staying strong and not revealing weakness are to Icelandic culture. As with many other individualistic countries, value is placed on independence and self-reliance [87,88,89]. Additionally, a new study examining cultural effects on trauma recovery processes of GBV survivors showed that fearing vulnerability and being perceived as weak were the most significant barriers among the American participants [90].

While the findings of this study are essential, they need to be considered alongside some limitations, such as the relatively small Qual and Quan samples selected using non-probability sampling methods. Probability sampling was unfeasible, as is true for many other studies among hidden and vulnerable populations [91,92]. Moreover, with the Qual data collection occurring before the Quan one, using identical or nested samples [71] was not possible, which can be a legitimation threat to the meta-inferences generated [57]. Still, legitimation strategies were applied to minimize these threats. Our samples were drawn from the same underlying population and comparable to most demographics collected. We had less information on the Quan sample, which is a limitation, and a noticeable difference between the samples was concerning age and prevalence of receiving mental healthcare. In addition, the Qual data collection was conducted before the COVID-19 pandemic, while the Quan data collection occurred in the first months of the pandemic in Iceland. The study’s cross-sectional design furthermore did not allow for longitudinal analysis of the relationships between the survivors’ distress symptoms, SOC, help-seeking barriers, and trauma recovery actions.

Moreover, we used a largely Quan approach to examine the divergent findings because we were trying to understand the issues revealed in the smaller Qual dataset in the larger Quan sample, allowing us to potentially generalize our findings. While balanced Quan and Qual sample sizes would be desirable, mixed methods research often has smaller Qual samples. What the mixing methods does, however, as demonstrated in this study, is use the findings from each to validate or legitimate the findings from the other. Our previous work had provided strong Qual evidence for the significance of these barriers [21,64,65], and therefore we decided to focus on the Quan data in this stage. The level of influence step was performed to gain information on the significance of the barriers from a Quan lens. As noted, these Quan data are cross-sectional, providing a point-in-time estimate, and additional Qual data can give us a story of a journey for deeper understanding. Research examining the help-seeking journey of Icelandic survivors is underway. Furthermore, it needs to be acknowledged that “mainstream” responses do not account for individual experiences, and the voices of those who have significantly different experiences of life to most of the population may be lost.

Finally, the work presented here illustrates that the factor analysis of requiring items to load only onto one factor in order to be considered “valid” may sometimes be troubling, promoting an overly reductionist approach that can oversimplify the complexities of social phenomena. A person’s view of the world might not align with the way instruments are developed, and we believe that the use of mixed methods, especially the legitimation strategy of integration illustrated here, can help move the field toward the critical examination of including participants’ voices in our instrument development and validation.

Future work on the construct validation of the Icelandic BHS-TR should further examine and develop the critical expansion and discordant items identified in this study. Additional interviewing with survivors to gain in-depth insights into the conceptual overlaps and cultural nuances of these barriers are needed to inform BHS-TR refinements further. In addition, assessing floor-and-ceiling effects for all items, examining the BHS-TR structure using confirmatory factor analysis, and evaluating response consistency via test–retest reliability measurement using large and representative samples is required. Finally, future studies with and about the BHS-TR would be greatly enhanced by utilizing recruitment procedures targeting diverse groups of survivors, including gender identity, ethnic background, socioeconomic status, forms of GBV, and help-seeking attempts or experiences. We hope the BHS-TR can serve as a valuable tool in future help-seeking research among survivors of IPV and other types of GBV. Along with creating the Icelandic BHS-TR, our research group, the Multicultural Study of Trauma Recovery network (https://mistory-traumarecovery.org/, accessed on 22 March 2022), is currently working on adapting the BHS-TR to various cultures and contexts.

## 5. Conclusions

The current study focused on mixed methods integration to inform validation evidence for a new help-seeking barriers instrument centered on trauma recovery. The overall findings indicated that the BHS-TR is a trustworthy and valid measure but deserves continuing attention for refinements. Expanding our measure based on the lived experience of survivors can help BHS-TR better capture the significant hindrances faced and the immense amount of effort survivors often take to seek help. Measuring the various barriers to seeking help for trauma recovery among survivors can move the IPV help-seeking literature into a more holistic and survivor-centered direction. This study adds to the growing literature supporting the benefits of using mixed methods for instrument development and validation. Moreover, its findings highlight the importance of giving attention to the voices of the target population throughout the validation process. Finally, the authors hope that the current study can serve as an exemplar to encourage mixed methods researchers faced with divergent findings to embrace the possibility of expanding insights from additional analyses and development of innovative follow-up strategies for a deeper, more nuanced understanding of complex phenomena.

## Figures and Tables

**Table 1:** Characteristics of participants in both phases.

Characteristics	Qual Phase (*n* = 17)	Quan Phase (*n* = 137)
Age		
18–29	4 (23.5%)	24 (17.5%)
30–39	7 (41.2%)	34 (24.8%)
40–49	4 (23.5%)	38 (27.7%)
50–59	1 (5.9%)	18 (13.1%)
60+	1 (5.9%)	6 (4.4%)
Not stated	-	17 (12.4%)
Racial and ethnic background		
Caucasian	17 (100%)	-
Iceland-born	16 (94.1%)	-
Foreign-born	1 (5.9%)	-
Level of education		
High school or less	3 (17.6%)	11 (8.0%)
Technical or junior college degree	5 (29.4%)	29 (21.2%)
University degree	9 (52.9%)	82 (59.9%)
Not stated	-	15 (10.9%)
Employment status (not mutually exclusive)		
Working	12 (70.6%)	88 (64.2%)
Unemployed or looking for work	2 (11.8%)	7 (5.1%)
Student	5 (29.4%)	26 (19.0%)
Homemaker	1 (5.9%)	3 (2.2%)
Unable to work due to sickness/disability	3 (17.6%)	20 (14.6%)
Other	-	24 (17.5%)
Number of children		
None	5 (29.4%)	24 (17.5%)
One or two	9 (52.9%)	59 (43.1%)
Three or more	3 (17.6%)	46 (33.6%)
Not stated	-	8 (5.8%)
Years in the abusive relationship		
1–5	4 (23.5%)	-
6–10	9 (52.9%)	-
11–15	2 (11.8%)	-
15+	2 (11.8%)	-
Years out of the abusive relationship		
1–5	10 (58.8%)	-
6–10	6 (35.3%)	-
11–15	1 (5.9%)	-
Current medical diagnosis (mental and/or physical)		
No	6 (35.3%)	44 (32.1%)
Yes	11 (64.7%)	93 (67.9%)
History of receiving mental healthcare		
No	8 (47.1%)	24 (17.5%)
Yes	9 (52.9%)	112 (81.8%)
Not stated	-	1 (0.7%)

**Table 2:** Joint display of the coherence of findings for structural barriers to help seeking.

Conceptual Structure	Qualitative Phase	Quantitative Phase	Coherence of Findings
Structural Barriers	In the interviews, participants (14 of 17) generally made a specific distinction between structural and internal barriers to seeking help. When discussing structural barriers, the women mainly mentioned system-level barriers referring to healthcare and social services. Findings provided evidence of relevance, face validity, and content validity. *“There are so many walls to climb over in our system, and when you are so shattered and exhausted, you just can’t.”*	The “Structural Barriers Index” included Financial Concerns, Unavailable/Not Helpful, External Constraints, and Inconvenience factors. The index had good internal consistency (α = 0.75), and the results provided evidence of convergent, discriminant, and known-groups validity.	Complementarity: Not included in subsequent analysis.
Financial Concerns	A majority (12 of 17) of the participants agreed that the items about financial concerns were significant barriers, especially related to seeking professional psychological help, as the Icelandic Health Insurance covers not all mental healthcare. Findings provided evidence of relevance, face validity, and content validity. *“Let’s be clear, getting professional help to work through your trauma is hardly part of our great welfare system, and I couldn’t even pay the bills, let alone go to a psychologist.”*	The “Financial Concerns” factor comprised items #2, 19, and 18. All items had high factor loadings, and the internal consistency was good (α = 0.82). Results provided evidence of convergent, discriminant, and known-groups validity.	Complementarity: Not included in subsequent analysis.
Unavailable/Not Helpful	While these items did not represent the main barriers hindering the women from seeking help, more than half (11 of 17) said that the healthcare they needed had not been available to them. Findings provided evidence of relevance, face validity, and content validity. *“I didn’t tick in the right boxes when I finally had the courage to go to the hospital. Like sure honey, we will stitch up your head … but you won’t get mental healthcare there.”*	The “Unavailable/Not Helpful” factor comprised items #15, 16, and 17. All items had high factor loadings, and the internal consistency was good (α = 0.71). Furthermore, results provided evidence of convergent, discriminant, and known-groups validity.	Complementarity: Not included in subsequent analysis.
External Constraints	Many (11 of 17) participants were afraid of the consequences of seeking help, and the other external constraints impacted them as well. Findings provided evidence of relevance, face validity, and content validity. *“You can lose so much … friends and family members who sided with him, and I knew he would use it against me in the custody battle … unfit mentally ill mother.”*	The “External Constraints” factor comprised items #14, 34, and 25. All items had high factor loadings, and the internal consistency was good (α = 0.77). Furthermore, results provided evidence of convergent, discriminant, and known-groups validity.	Complementarity: Not included in subsequent analysis.
Inconvenience	The inconveniences barriers were not the foremost reasons stopping participants from seeking help. However, a majority (10 of 17) thought these barriers were part of the picture. The most mentioned was the time factor. Findings provided evidence of relevance, face validity, and content validity. *“There was no time … being a single mom working a full-time job doesn’t give you a lot of space.”*	The “Inconvenience” factor was comprised of items #5 and 8. The items had high factor loadings, but the internal consistency was poor (α = 0.52). Evidence of convergent, discriminant, and known-groups validity was provided. One inconvenience item (#9) about not getting time away from work or family needed to be dropped as it did not load significantly onto this or any other factor.	Expansion: Item #9 was included in the subsequent analysis.
Discrimination	The participants interpreted the prejudice and discrimination items as relating to race and ethnic background, which did not apply to them but recognized these items would be important for the survivor immigrants to Iceland. Yet, many (13 of 17) said they were worried about and experienced prejudice and discrimination for being an IPV survivor. These experiences centered around stereotyping and victim-blaming. *“Take the risk of revealing myself as a victim … no. When people know your story, it’s like you become nothing else, the weak abused women stamp is burnt to your forehead.”*	All the discrimination items (#20, 21, and 23) were identified as problematic due to cross-loadings onto different factors and needed thus to be dropped.	Expansion: Items #20, 21, and 23 were included in subsequent analysis.

Notes: Qualitative findings were generated using deductive and inductive qualitative content analysis; quantitative results were generated using principal component analysis, multidimensional scaling, Cronbach’s alpha coefficient (α), Pearson’s correlation coefficient, and independent sample *t*-tests.

**Table 3:** Joint display of the coherence of findings for internal barriers to help seeking.

Conceptual Structure	Qualitative Phase	Quantitative Phase	Coherence of Findings
Internal Barriers	Most participants (14 of 17) distinguished between structural and internal barriers to seeking help. They understood that internal barriers arose from internalized beliefs or values and personally held fears. Findings provided evidence of relevance, face validity, and content validity. *“When these beliefs and attitudes are everywhere in the society, your family, you grew up around this mentality, of course, you start to believe it yourself.”*	The “Internal Barriers Index” included the Weakness/Vulnerability, Problem Management Beliefs, Frozen/Confused, and Shame factors. The index had good internal consistency (α = 0.88), and the results provided evidence of convergent, discriminant, and known-groups validity.	Complementarity: Not included in subsequent analysis.
Reveals Weakness	All of the participants (17 of 17) said that help-seeking and being vulnerable felt like a sign of weakness. These beliefs were significant deterrents to seeking help but were identified as missing from the scale. Four new items reflecting aspects of this category were developed, and findings provided evidence of relevance, face validity, and content validity. *“It was so strong within me the need to be tough and keep going, needing help felt like a sign of weakness.”*	The “Weakness/Vulnerability” factor was comprised of items #40, 39, 35, 41, and 24. Of these items, three were new revealing weakness items, one new safeguard item, and one shame item. All had high factor loadings, and the internal consistency was good (α = 0.86). Furthermore, results provided evidence of convergent, discriminant, and known-groups validity. One weakness item about the vulnerability of opening up to your feelings (#38) needed to be dropped as it significantly loaded onto another factor as well.	Expansion: Item #38 was included in the subsequent analysis.
Problem Management Beliefs	Most (14 of 17) participants said the problem management beliefs items accurately described their coping. Findings provided evidence of relevance, face validity, and content validity. *“Definitely didn’t think it was serious enough to seek professional help and like with others … you are supposed to be able to, and I just really wanted to deal with it myself.”*	The “Problem Management Beliefs” factor comprised items #1, 11, and 10. All items had high factor loadings, and the internal consistency was fair (α = 0.62). Furthermore, results provided evidence of convergent, discriminant, and known-groups validity.	Complementarity: Not included in subsequent analysis.
Frozen/ Confused	All participants had experienced being frozen and confused, and many (13 of 17) strongly agreed that this hindered seeking help. Findings provided evidence of relevance, face validity, and content validity. *“I couldn’t think straight and felt like I couldn’t move, you know this emotional numbness is so hindering.”*	The “Frozen/Confused” factor comprised items #29, 30, 26, and 27. All items had high factor loadings, and the internal consistency was good (α = 0.79). Furthermore, results provided evidence of convergent, discriminant, and known-groups validity.	Complementarity: Not included in subsequent analysis.
Shame	All participants endorsed shame, and most (15 of 17) talked about many layers of shame as a primary barrier. Findings provided evidence of relevance, face validity, and content validity. *“I was racked with shame. For being so stupid of getting myself into this. For allowing him to break me. For staying. For whom I had become.”*	The “Shame” factor comprised items #6, 7, and 28. All items had high factor loadings, and the internal consistency was good (α = 0.83). Furthermore, results provided evidence of convergent, discriminant, and known-groups validity.	Complementarity: Not included in subsequent analysis.
Mistrust/ Rejection	Mistrust and perceived rejection of people or systems were prominent barriers in the participants’ narratives (14 of 17) when discussing these items and often connected to their former attempts to seek help. Findings provided evidence of relevance, face validity, and content validity. *“I didn’t trust anyone, always scared of being betrayed, and like the lack of understanding I got from my family, it took me so many steps back, years until I tried to seek help again.”*	The mistrust and rejection items (#31, 32, and 33) were identified as problematic due to cross-loadings onto different factors.	Discordance: Items #31, 32, and 33 were included in the subsequent analysis.
Safeguard Yourself	Most (15 of 17) participants described the desire and efforts to protect themselves from further pain. These help-seeking barriers had strongly influenced them but were identified as missing from the scale. Three new items reflecting aspects of this category were developed, and findings provided evidence of relevance, face validity, and content validity. *“I wanted to protect myself, and I was in this mode that I just could not deal with it and needed to let myself be there, but […] you can get stuck.”*	The safeguard items together did not make a new factor. Two of the items (#36 and 37) needed to be dropped as they cross-loaded. Item #41 belonged to the “Weakness/Vulnerability” factor.	Discordance: Items #36 and 37 were included in the subsequent analysis.

Notes: Qualitative findings were generated using deductive and inductive qualitative content analysis; quantitative results were generated using principal component analysis, multidimensional scaling, Cronbach’s alpha coefficient (α), Pearson’s correlation coefficient, and independent sample *t*-tests.

**Table 4:** Ranking of the items after influence (*n* = 137).

Item Number	BHS-TR Item (Index: Subscale)	M	SD	*n* (%) *
7	I was ashamed (Internal: Shame)	3.21	1.00	106 (77.4%)
29	I could not seem to clarify my feelings or know what I needed (Internal: Frozen/Confused)	3.15	0.90	110 (80.3%)
10	I wanted to or thought I should solve the problems on my own (Internal: Problem Management Beliefs)	3.09	1.03	104 (75.9%)
1	I thought the problem would probably get better by itself (Internal: Problem Management Beliefs)	2.99	1.03	104 (75.9%)
41	Seeking help would require acknowledging things I did not want to face (Internal: Weakness/Vulnerability)	2.84	1.23	87 (63.5%)
26	I was confused or unable to plan out all the details or steps (Internal: Frozen/Confused)	2.82	1.10	89 (65.0%)
11	I thought the situation was normal or was not severe (Internal: Problem Management Beliefs)	2.79	1.13	93 (67.8%)
**38**	**I felt like opening up to my feelings would weaken me** (Dropped)	2.78	1.11	86 (62.8%)
30	I was afraid I could not clearly express what I needed (Internal: Frozen/Confused)	2.75	1.12	84 (61.3%)
39	Getting help would mean that I had failed or had been defeated (Internal: Weakness/Vulnerability)	2.74	1.16	83 (60.6%)
27	I felt paralyzed or frozen and unable to get started (Internal: Frozen/Confused)	2.66	1.24	79 (57.7%)
2	I was concerned that the help I needed would be too expensive (Structural: Financial Concerns)	2.65	1.12	79 (57.7%)
28	I believed that people would judge me (Internal: Shame)	2.61	1.17	70 (51.1%)
40	I thought that strong people should not need help (Internal: Weakness/Vulnerability)	2.60	1.21	78 (56.9%)
24	I thought the situation was too personal or wanted to keep it private (Internal: Weakness/Vulnerability)	2.58	1.19	79 (57.7%)
**33**	**I felt no one could understand or help me** (Dropped)	2.58	1.13	76 (55.5%)
6	I was concerned about what others might think (Internal: Shame)	2.54	1.20	75 (54.7%)
**32**	**I felt that I could not trust people to help me** (Dropped)	2.46	1.04	73 (53.8%)
**36**	**I was afraid that seeking help would be** **too emotionally difficult or hurt me even more** (Dropped)	2.42	1.15	72 (52.6%)
**37**	**I did not seek help in an effort to protect or safeguard myself** (Dropped)	2.40	1.08	70 (51.1%)
35	I was scared of being seen as weak (Internal: Weakness/Vulnerability)	2.39	1.13	64 (46.7%)
25	I was afraid of the consequences for myself, my children, or my family (Structural: External Constraints)	2.37	1.25	64 (46.7%)
18	I did not have adequate financial resources (Structural: Financial Concerns)	2.36	1.22	66 (48.2%)
8	I thought getting help would take too much time or was inconvenient (Structural: Inconvenience)	2.32	1.13	60 (43.8%)
**31**	**I was afraid I would explain what I needed, and no one would help me anyway** (Dropped)	2.23	1.13	57 (41.6%)
19	The available health insurance would not cover the type of treatment I needed (Structural: Financial Concerns)	2.13	1.25	53 (38.7%)
**23**	**I felt that there would be prejudice or discrimination against me** (Dropped)	2.07	1.15	49 (35.8%)

Notes: The influential dropped items are indicated in bold; M = mean; SD = standard deviation; * frequency and percent endorsed as somewhat or strongly influenced me.

**Table 5:** Cross-loadings of the dropped items.

Dropped Items	BHS-TR Factors (Loadings)	Inferences
**Mistrust/Rejection**		
I felt no one could understand or help me (#33)	Frozen/Confused (−0.41) Unavailable/Not Helpful (0.37)	The mistrust items were cross-loaded with Frozen/Confused (internal) items and structural items, either Unavailable/Not Helpful or External Constraints. Despite this cross-loading with being frozen, the fear of rejection is the cause of the freezing. Moreover, while it may be related to the structural barriers, they do not capture the concept of mistrust and perceived rejection. We believe that the wording of these items does not adequately capture the social component of the “mistrust” concept. The concept includes betrayal, rejection, stigma, problems with past encounters, the general feeling of being misunderstood by society and people in the service sector. This factor requires more analysis and evaluation.
I was afraid I would explain what I needed, and no one would help me anyway (#31)	Frozen/Confused (0.40) External Constraints (0.36)	
I felt that I could not trust people to help me (#32)	Unavailable/Not Helpful (0.43) Frozen/Confused (0.39)	
**Safeguard Yourself**		
I did not seek help in an effort to protect or safeguard myself (#37)	Inconvenience (0.53) Weakness/Vulnerability (0.52)	Both safeguarding items were cross-loaded onto the Weakness/Vulnerability (internal) and Inconvenience (structural) factors. We believe that neither of these factors captures the safeguarding efforts that the items were intended to measure. These loadings indicate a problem with the wording of the items, and revisions are needed. The former item might need to be more specific about safeguarding from what. The common thread underlying the development of the initial category was safeguarding from being more hurt, which had many aspects. One frequently mentioned was protecting from not being believed, which may be desirable to make into a specific item. *“You know if I would seek help, and I wouldn’t be* *believed, or it wouldn’t be taken seriously, I was* *dealing with enough.”* On the other hand, the second item might be too packed and possibly double-barreled. It was developed based on the commonly mentioned fear of re-traumatization by seeking help, still using the women’s words. *“I had made my world trigger-free, so yeah, I was* *really isolated, but […] to go there, talking about it would only hurt me even more.”* A potential change is to take out the ‘too emotionally difficult’ part. Further examination is needed to inform revisions of both items.
I was afraid that seeking help would be too emotionally difficult or hurt me even more (#36)	Weakness/Vulnerability (0.56) Inconvenience (0.50)	
**Reveals Weakness**		
I felt like opening up to my feelings would weaken me (#38)	Weakness/Vulnerability (0.44) Shame (0.43)	The weakness item loaded onto two internal factors at about the same strength. While the Weakness/Vulnerability and Shame factors are related, we believe they are conceptually distinct. In the interviews’, showing weakness was considered shameful. The narratives behind the development of this specific item were indeed related to being ashamed if they opened up and how that made them feel “less-than.” *“It was like I would somehow become less … I don’t like being vulnerable […] a big part of seeking help.”* For now, this item should stay as it stands but deserves further evaluation.
**Discrimination**		
I felt that there would be prejudice or discrimination against me (#23)	Shame (0.59) Discrimination (0.46)	The discrimination (structural) item loaded more strongly on the Shame (internal) factor in our sample, which supports the necessity for expanding the Discrimination factor. This item as it stands needs to be clarified to reference race, ethnicity, religion, or language, and new GBV specific prejudice/stigma item(s) need to be developed.

**Table 6:** Known-groups validity results on the item level.

BHS-TR	Depression			PTSD			SOC		
Dropped Items	No (*n* = 80)	Probable (*n* = 57)	*p*	No (*n* = 75)	Probable (*n* = 62)	*p*	M/High (*n* = 51)	Low (*n* = 81)	*p*
**Mistrust/Rejection**									
I felt no one could understand or help me (#33)	2.35 (1.15)	2.88 (1.03)	0.01	2.36 (1.11)	2.82 (1.10)	0.02	2.29 (1.12)	2.75 (1.12)	0.02
I was afraid I would explain what I needed, and no one would help me anyway (#31)	2.00 (1.13)	2.54 (1.05)	0.00	2.04 (1.12)	2.45 (1.09)	0.03	2.00 (1.09)	2.39 (1.11)	0.05
I felt that I could not trust people to help me (#32)	2.21 (1.14)	2.80 (1.03)	0.00	2.20 (1.12)	2.75 (1.08)	0.00	2.08 (1.10)	2.70 (1.10)	0.00
**Safeguard Yourself**									
I did not seek help in an effort to protect or safeguard myself (#37)	2.14 (1.00)	2.68 (1.10)	0.00	2.03 (0.97)	2.76 (1.07)	0.00	2.02 (1.02)	2.58 (1.08)	0.00
I was afraid that seeking help would be too emotionally difficult or hurt me even more (#36)	2.26 (1.16)	2.65 (1.11)	0.05	2.16 (1.13)	2.74 (1.10)	0.00	2.16 (1.19)	2.60 (1.11)	0.03
**Reveals Weakness**									
I felt like opening up to my feelings would weaken me (#38)	2.71 (1.08)	2.91 (1.14)	-	2.66 (1.11)	2.95 (1.09)	-	2.62 (1.07)	2.95 (1.11)	-
**Discrimination**									
I felt that there would be prejudice or discrimination against me (#23)	1.86 (1.09)	2.36 (1.18)	0.01	1.92 (1.17)	2.25 (1.12)	-	1.96 (1.13)	2.14 (1.19)	-

Notes: Independent sample *t*-tests; mean score (standard deviation); significance level at *p* ≤ 0.05; Patient Health Questionnaire-8 cut-off score of ≥10 for probable depression; Post-Traumatic Stress Disorder (PTSD) Checklist for DSM-5 cut-off score of ≥31 for probable PTSD; Sense of Coherence (SOC) Scale-13 scores of 13–57 for low SOC and scores of 58–91 for medium (M) to high SOC.

## Data Availability

The data presented in this study are available on reasonable request from the corresponding author. The data are not publicly available due to ethical and privacy reasons.

## Supplementary Materials

The following supporting information can be downloaded at: https://www.mdpi.com/article/10.3390/ijerph19074297/s1, Table S1: The Barriers to Help-Seeking for Trauma (BHS-TR) scale; Table S2: Items removed from the BHS-TR scale.
