# Supplementary material for: Using Mixed Methods Integration to Evaluate the Structure of Help-Seeking Barriers Scale: A Survivor-Centered Approach
Source: Int J Environ Res Public Health. 2022 Apr 3;19(7):4297. doi: 10.3390/ijerph19074297 (PMC8998269; doi:10.3390/ijerph19074297)
Supplement: Supplementary file 1 [file ijerph-19-04297-s001.zip › ijerph-1631711-supplementary.pdf]

Supplementary Materials

Supplementary Table S1. The Barriers to Help-Seeking for Trauma (BHS-TR) scale.

BHS-TR scale
Think about your experiences and feelings that are a result of gender-based violence. In the last 12 months, how much did the following reasons influence you in not seeking help?<br>(1) Did not influence me<br>(2) Influenced me a little<br>(3) Influenced me somewhat<br>(4) Strongly influenced me
Structural Barriers Index
**Financial Concerns**
2. I was concerned that the help I needed would be too expensive
19. The available health insurance would not cover the type of treatment I needed
18. I did not have adequate financial resources
**Unavailable/Not Helpful**
15. I was not satisfied with the available services
16. I felt that the help available would not provide the type of treatment or help that was best for the problem
17. I had sought help before, but it did not help
**External Constraints**
14. I was worried that if others discovered my health problems or situation, I could lose my children, security, or housing
34. Others were preventing me from getting the help I needed
25. I was afraid of the consequences for myself, my children, or my family
**Inconvenience**
5. I had distance or transportation problems
8. I thought getting help would take too much time or was inconvenient
Internal Barriers Index
**Weakness/Vulnerability**
40. I thought that strong people should not need help
39. Getting help would mean that I had failed or had been defeated
35. I was scared of being seen as weak
41. Seeking help would require acknowledging things I did not want to face
24. I thought my situation was too personal or wanted to keep it private
**Problem Management Beliefs**
1. I thought the problem would probably get better by itself
11. I thought the situation was normal or was not severe
10. I wanted to or thought I should solve the problems on my own
**Frozen/Confused**
29. I could not seem to clarify my feelings or know what I needed
30. I was afraid I could not clearly express what I needed
26. I was confused or unable to plan out all the details or steps
27. I felt paralyzed or frozen and unable to get started
**Shame**
6. I was concerned about what others might think
7. I was ashamed
28. I believed that people would judge me

Supplementary Table S2. Items removed from the BHS-TR scale.

BHS-TR dropped items
**From the original mental healthcare scale**
3. I was unsure about where to go for help or how to access help
4. I thought help probably would not do any good
9. I could not get time away from work or my family
12. I was concerned that I would not be able to get help soon enough
13. I was scared about being put into a hospital against my will
20. I felt that my culture, background, or specific situation would not be understood
21. Suitable professionals were not available to me
22. The kind of help I needed was not available
23. I felt that there would be prejudice or discrimination against me
**From the trauma-specific additions**
31. I was afraid I would explain what I needed, and no one would help me anyway
32. I felt that I could not trust people to help me
33. I felt no one could understand or help me
**From the cognitive interviews (emic) additions**
36. I was afraid that seeking help would be too emotionally difficult or hurt me even more
37. I did not seek help in an effort to protect or safeguard myself
38. I felt like opening up to my feelings would weaken me
